# Comparative evaluation of effect of sodium hypochlorite and chlorhexidine in dental unit waterline on aerosolized bacteria generated during dental treatment

**DOI:** 10.1186/s12903-023-03585-9

**Published:** 2023-11-14

**Authors:** Rutuja Patil, Ajit Hindlekar, Ganesh R. Jadhav, Priya Mittal, Vamshi Humnabad, Marco Di Blasio, Marco Cicciù, Giuseppe Minervini

**Affiliations:** 1 Department of Conservative Dentistry and Endodontics, Dr D. Y. Patil Dental College & Hospital, Dr. D. Y. Patil Vidyapeeth, Pimpri, Pune -18 India; 2 Dept of Dentistry, AIIMS Nagpur, Nagpur, India; 3 Department of Conservative Dentistry and Endodontics, Swargiya Dadasaheb Kalmegh Smruti Dental College & Hospital, Nagpur, India; 4 Department of Medicine and Surgery, University Center of Dentistry, University of Parma, 43126 Parma, Italy; 5 Department of Biomedical and Surgical and Biomedical Sciences, Catania University, 95123 Catania, Italy; 6 Saveetha Dental College & Hospitals, Saveetha Institute of Medical & Technical Sciences Saveetha University, Chennai, India; 7 Multidisciplinary Department of Medical-Surgical and Odontostomatological Specialties, University of Campania “Luigi Vanvitelli”, Naples, Italy

**Keywords:** Agar plates, Chlorhexidine, Dental aerosols, Dental unit waterline, Sodium hypochlorite

## Abstract

**Background:**

In dentistry, nosocomial infection poses a great challenge to clinicians. The microbial contamination of water in dental unit waterlines (DUWLs) is ubiquitous. Such infected DUWLs can transmit oral microbes in the form of aerosols. Previous studies have suggested treating DUWLs with various disinfectants to reduce cross-contamination. The literature lacks a comparative evaluation of the effect of the use of 0.2% chlorhexidine (CHX) and 0.1% sodium hypochlorite (NaOCl) in DUWLs on aerosolized bacteria generated during dental procedures.

**Objective:**

To compare the effect of NaOCl and CHX in DUWLs on aerosolized bacteria generated during restorative and endodontic procedures.

**Materials and methods:**

A total of 132 patients were equally divided into three groups (*n* = 44 in each group) according to the content of DUWL as follows.

Group I—0.1% NaOCl

Group II—0.2% CHX

Group III—distilled water (Positive control)

One-way ANOVA was performed and the Kruskal–Wallis test was used for intergroup comparison.

**Results:**

For the restorative procedure, inter-group comparison of mean colony-forming units (CFU) scores showed a statistically significant difference between the groups (*p* - .001) with the score of group 3 higher than group 2 followed by group 1. For the endodontics, an inter-group comparison of CFU scores showed a statistically significant difference between the groups (*p* - .003) with the mean score in group 1 being the lowest and group 3 being the highest.

**Conclusion:**

The addition of NaOCl or CHX in DUWLs shows an effective reduction in aerosolized bacteria compared to distilled water.

## Introduction

In dentistry, nosocomial infection, which is acquired during dental treatment, poses a great challenge to clinicians [1–5]. Despite several efforts to avoid such cross-infections, there is an increased risk of spread of infection during dental treatment as the same dental chair set-up is used to treat several patients. The nature of these infections can be bacterial, viral, or fungal. The most commonly used part of a dental chair is a high-speed handpiece which is based on a compressed air-driven mechanism and needs a constant supply of running water for cooling [6, 7]. Aerosol develops during all dental manoeuvres, especially during prosthesis [8–13].This cooling is achieved through a dental water supply system by the intricate network of organized fine‐diameter plastic tubes. This complex system is called a dental unit waterline (DUWLs).

Various studies have stated that the microbial contamination of water in dental unit waterlines (DUWLs) is ubiquitous [14–18]. These microbes mainly come from the water supply or back-siphonage of oral fluids into the DUWLs during the treatment. Moreover, various factors such as water stagnation, laminar water flow, and anti-retraction valve failure in handpieces favour the surface colonization and replication of microbes [19, 20]. It results in adherent heterogeneous microbial accumulations termed “biofilm” [19, 21]. Thus, DUWLs provide the place for the growth and maturation of biofilm of mesophilic, heterotrophic and aerobic microorganisms. Such contaminated DUWLs cause great risk to dental healthcare staff and patients with systemic conditions like diabetes, HIV-positive, chronic alcoholics, smokers, immunocompromised patients etc. [22, 23].

Aerosols as well as spatter generated during dental procedures can transmit microbes from the oral cavity and airway tract [24]. Hence it can contaminate the skin and mucous membrane of the mouth, respiratory passages and eyes of dental personnel or other patients. Such dental aerosols (DAs) are composed of liquid or solid particles of a size of less than 50 µm, which can become airborne and can be transmitted to a considerable distance [25]. Apart from microorganisms (such as bacteria, viruses and protozoa) and metabolites (such as endotoxins and toxins), they may also contain components of saliva and blood thus possessing a considerable risk for the spread of nosocomial infection for the treating dentist as well as other patients [26]. They have the ability to remain suspended in the air for hours before entering the respiratory tract and reaching down the alveolar spaces. High-speed handpiece use can cause dental aerosols (DAs) to travel a significant distance of 1.5 m with high concentrations [27]. The generation of such infectious DAs directed the Occupational Safety and Health Administration to classify dentistry as a “very high-risk” profession [28].

American Dental Association and the Centers for Disease Control and Prevention have set a limit of < 200 CFU/ml for maximum microbial load delivered by DUWLs [29]. As a result, timely flushing of waterlines, independent water reservoir systems, use of distilled or electrolyzed water, ultraviolet light, micropore filtration, and intermittent or continuous chemical disinfection etc. have been developed to decrease microbial growth and colonization [30–34]. Many studies have suggested the treatment DUWLs with various disinfectant solutions, including povidone-iodine, chlorhexidine gluconate, sodium hypochlorite, hydrogen peroxide, Poseidon-S, nanometer silver disinfectant etc. [35–47]. Chlorhexidine gluconate (CHX) is an FDA-approved, antimicrobial bis-biguanide showing efficacy against a broad spectrum of bacteria as well as fungus by disrupting the cell membrane [48–50]. Sodium hypochlorite (NaOCl) demonstrates not only antibacterial but also virucidal and fungicidal activity by action on sulphydryl groups of bacterial enzymes through oxidative mechanism [51]. Various studies have used either NaOCl or CHX in DUWLs. The literature lacks a comparative evaluation of the effect of the use of 0.2% CHX and 0.1% NaOCl in DUWLs on aerosolized bacteria generated. Moreover, DAs produced during nonsurgical endodontic treatment (NSRCT) and restorative procedures (RP) were not compared in any of the previous studies. Hence this study was planned and conducted with primary objective to evaluate whether there is any difference in the aerosolized bacteria generated during nonsurgical endodontic treatment (NSRCT) and restorative procedures (RP). The secondary objective of the study was to compare the effect of NaOCl and CHX in DUWLs on aerosolized bacteria generated during RP and NSRCT. The first part of null hypothesis assumes that there is no generation of aerosolized bacteria during these dental procedures and second part assumes that the addition of NaOCl as well as CHX in DUWLs does not show any effect on it. The alternate hypothesis is that aerosolized bacteria are generated during these dental procedures and addition of NaOCl—CHX in DUWLs affects it.

## Materials and method

This study was initiated after getting approval from the institutional ethics committee of Dr. Y.Patil Dental College and Hospital, Pimpri, Pune (5/11/75/XX/IEC/2021/X). The study was carried out in accordance with the Helsinki Declaration of 1975, as revised in 2000. Informed consent was obtained from all the participants and/or legal guardians for the study. The sample size was calculated using G*Power 3.1.9.4 software. Keeping the α error of 6%, the power of the study at 80% and the effect size of 0.5, the sample size was found to be 132. Hence total of 132 patients needing either class I restorative procedure (*n* = 66) or endodontic treatment (*n* = 66) were recruited in the study. These patients were equally divided into three groups (*n* = 44 in each group) according to the content of DUWL into three separate dental operatories (1, 2, and 3) as follows.

- Group I (*n* = 22 for RP and *n* = 22 for NSRCT)—0.1% sodium hypochlorite (NaOCl, Prime Dental, India) in DUWL of dental operatory 1
- Group II (*n* = 22 for RP and *n* = 22 for NSRCT)—0.2% chlorhexidine (CHX) (CHLOR X, Prevest Denpro, India) in DUWL of dental operatory 2
- Group III (*n* = 22 for RP and *n* = 22 for NSRCT)—distilled water in DUWL of dental operatory 3 (Positive control)

**Fig. 1:**
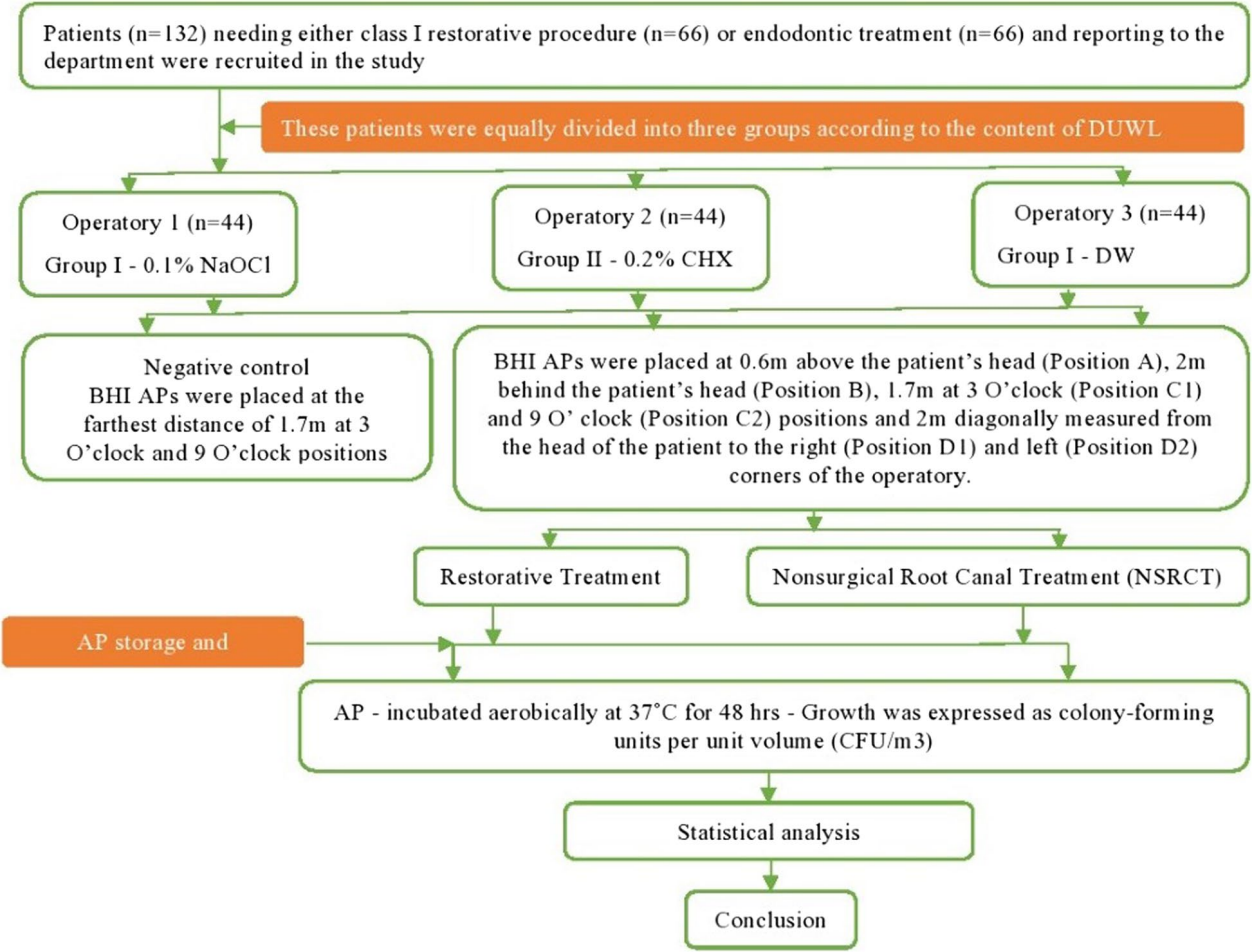
Flowchart depicting all the steps of the current study

Three dental operatories with self‑contained water reservoirs were selected for the study from the institute's Conservative Dentistry and Endodontics Department. Using environment-protected agency-registered disinfectant, the exposed surfaces of all three operatories were cleaned and disinfected. All the steps of the current study are mentioned in a flowchart (Fig. 1).

### Placement of agar plates for baseline sample collection (Negative control)

**Fig. 2:**
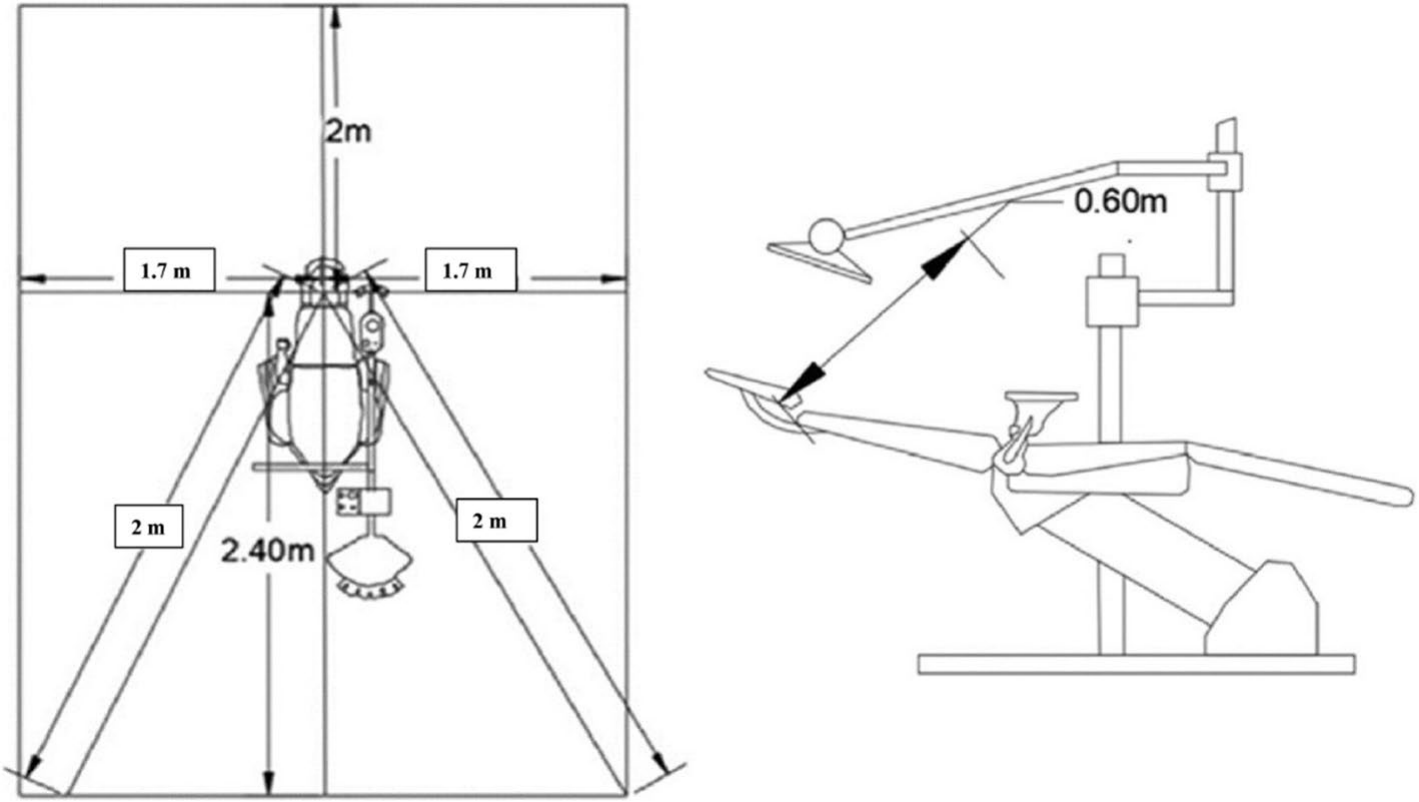
Shows blood-supplemented brain–heart infusion (BHI) 10 cm^2^ Agar Plates (AP) were placed at 0.6 m above the patient's head, 2 m behind the patient's head, 1.7 m at 3 O'clock and 9 O'clock positions and 2 m diagonally measured from the head of the patient to the right and left corners of the operatory

For the baseline sample collection (negative control), blood-supplemented brain–heart infusion (BHI) 10 cm^2^ Agar Plates (AP) (Bihangam, India) were placed at the farthest distance of 1.7 m at 3 O’clock and 9 O’clock positions all three dental operatories (Fig. 2). After 24 h, Agar Plates (AP) were sent for evaluation of bacterial growth. During that period, no procedure was performed in all dental operatories.

### Placement of agar plates for distilled water in DUWL of dental operatory 3 (Group III—Positive control)

**Fig. 3:**
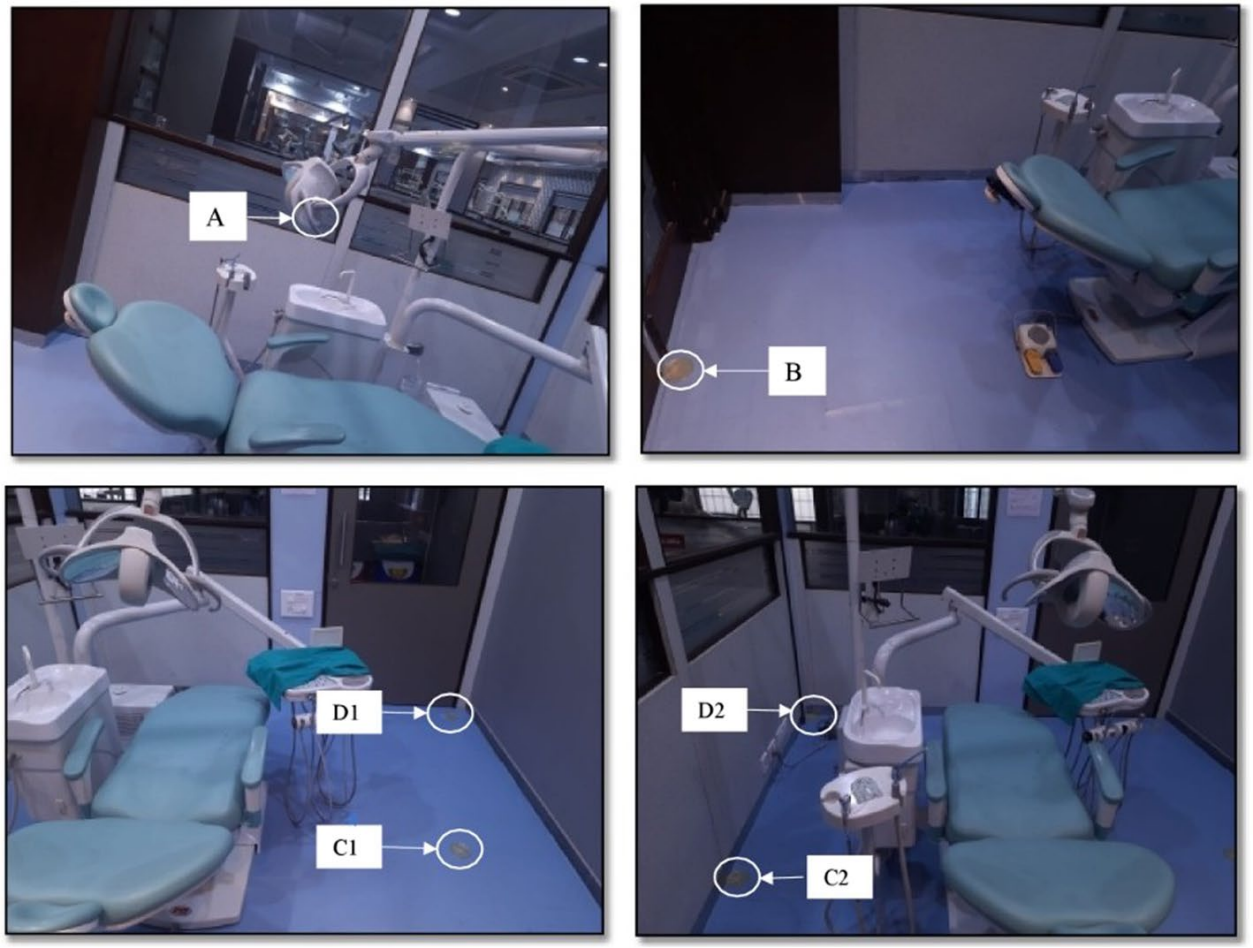
Shows blood-supplemented brain–heart infusion (BHI) 10 cm Agar Plates. (AP) were placed at 0.6 m above the patient's head (**A**), 2 m behind the patient's head. (**B**), 1.7 m at 3 O'clock (C1) and 9 O'clock (C2) positions and 2 m diagonally measured from the head of the patient to the right (DI) and left (D2) corners of the operatory

The positive control sample was measured by filling a dental operatory water bottle with distilled water. For evaluating the generation and extent of DAs dissemination, Blood-supplemented brain–heart infusion (BHI) AP were placed at the predetermined positions using double coated urethane foam tapes (3 M, Minnesota, U.S). BHI APs were placed at 0.6 m above the patient’s head (Position A), 2 m behind the patient’s head (Position B), 1.7 m at 3 O’clock (Position C1) and 9 O’ clock (Position C2) positions and 2 m diagonally measured from the head of the patient to the right (Position D1) and left (Position D2) corners of the operatory (Fig. 3). Dental unit waterlines were flushed before the treatment procedure. The patients were asked to pre-rinse with 1.5% povidone-iodine mouthwash before the start of treatment. All the treatments were carried out under rubber dam isolation.

#### Endodontic Treatment – Nonsurgical Root Canal Treatment (NSRCT)

The molars needing root canal treatment due to carious exposure with delayed response to pulp sensibility tests and vital pulp on entering the pulp chamber were included in the study. A 2% ilidocaine with i1:80000 epinephrine (Xicaine, ICPA Health Products Ltd., Mumbai, India) was used toiadminister the IANB using the traditional Halsted technique. In a closed operatory 3, access cavity preparation was initiated using high-speed air rotar (NSK, Japan) using round carbide bur (Mani Japan) and endo Z safe end bur (Dentsply, USA).

#### Restorative Procedure (RP)

For the restorative procedure patients with class 1 cavities were selected. The cavity was made with the help of a no. 245 carbide bur (Mani, Japan).

All endodontic and restorative treatments were completed using a standard protocol.

### Placement of agar plates for NaOCl in DUWL of dental operatory 1 (Group I)

For a 6% NaOCl solution, 1:60 dilution was done with a measuring cylinder to make 0.1% NaOCl for DUWL. The water bottle of dental operatory 1 was filled with 0.1% NaOCl and the rest of the procedure was done the same as in operatory 3.

### Placement of agar plates for CHX in DUWL of dental operatory 2 (Group II)

The water bottle of dental operatory 1 was filled with 0.2% CHX and the rest of the procedure was done the same as in operatory 3.

All the procedures were carried out within 90 mins under the same clinical conditions.

### Agar sample storage and processing

**Fig. 4:**
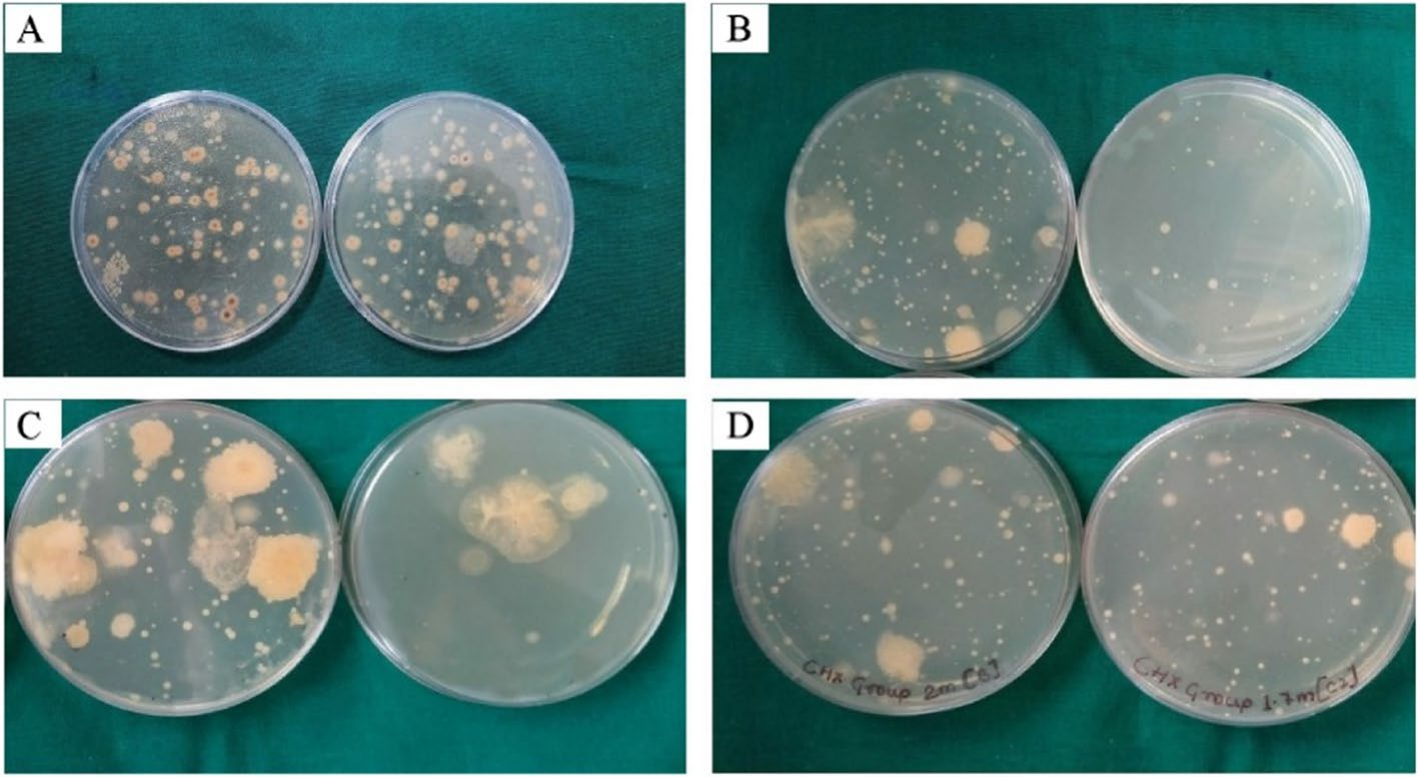
Shows blood-supplemented brain–heart infusion (BHI) 10 cm? Agar Plates (AP) showing bacterial growth as expressed as colony-forming units per unit volume (CFU/m3) in baseline (**A**), group I (**B**), group I (**C**), and group III (**D**)

**Fig. 5:**
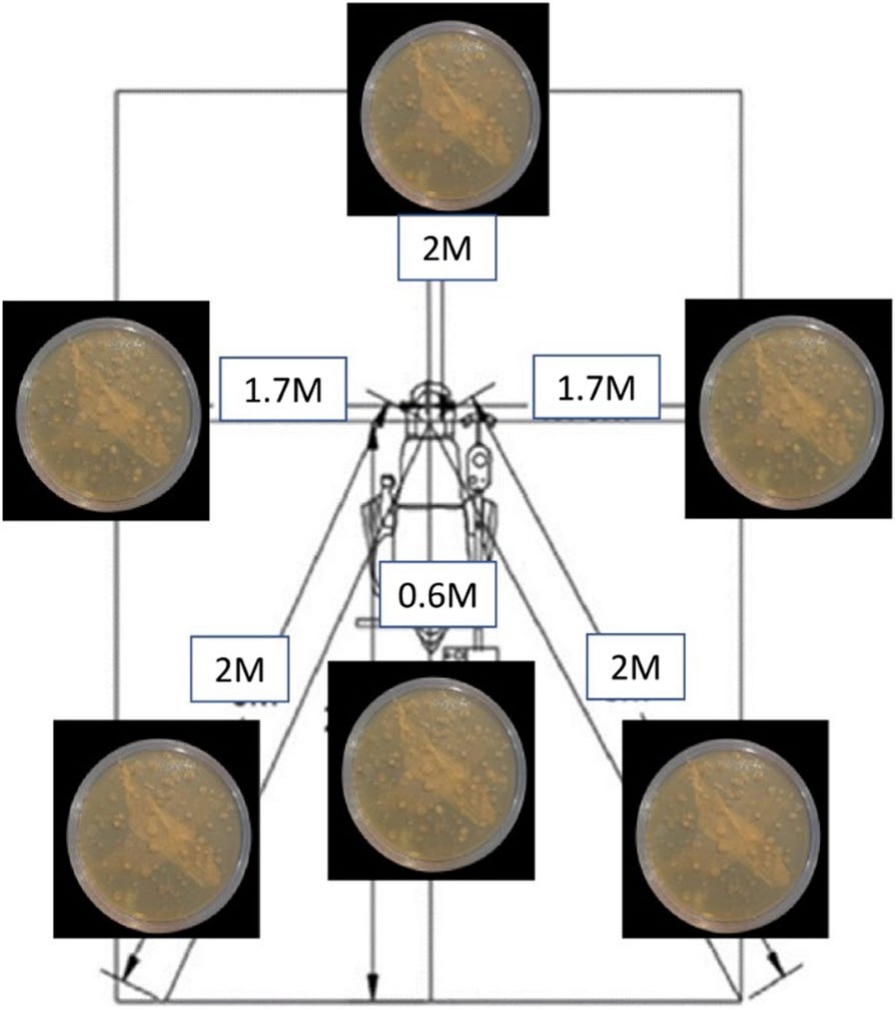
Shows variation in bacterial growth as expressed by colony-forming units per unit volume (CFU/m3) according to various distances from aerosols-producing source

In a non-sealed sterilization pouch exposed agar plates were placed and taken immediately to the laboratory for processing. Incubation of plates was done aerobically at 37˚C for 48 h. Growth was expressed as colony-forming units per unit volume (CFU/m3) as described in previous studies (Figs. 4 and 5).

## Results

**Fig. 6:**
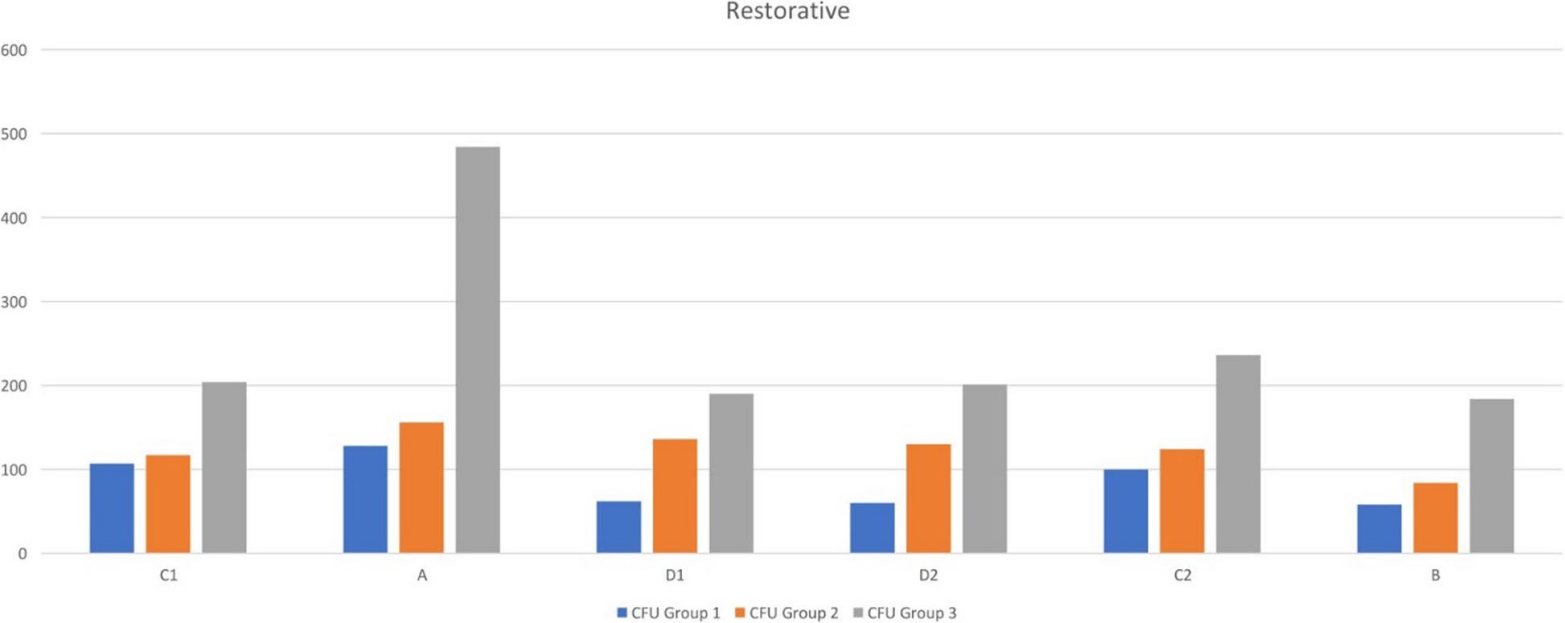
Shows the graph of the average values of CFU counts in operatories 1,2, and 3 when distilled water, NaOCI, and CHX were used in DUWL at all six positions during class I cavity preparation

**Fig. 7:**
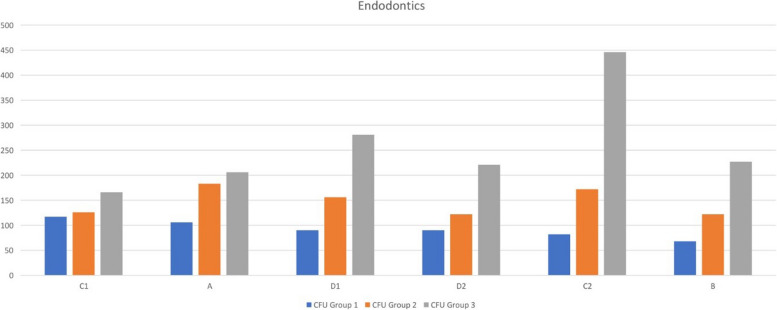
Shows the graph of the average values of CFU counts in operatories 1,2, and 3 when distilled water, NaOCI, and CHX was used in DUWL at all six positions during root canal treatment

The microbial load present preoperatively was evaluated using a passive sampling technique (negative control) in all three operatories (*n* = 6). Our data demonstrate that the pre-operative baseline CFU counts in operatories 1 (129.50), 2 (130.50), and 3 (130.00) were comparable (Table 1). The cumulative data of mean CFU score at six positions in all three operatories for RP (*n* = 22 in each operatory) and NSRCT (*n* = 22 in each operatory) are represented in the form of graphs in Figs. 6 and 7 respectively. Data from the current study showed that the NSRCT and restorative procedures led to the production of DAs as seen by the presence of increased CFU in the growth media of Operatory 3 (positive control). Use of distilled water generated the highest number of DAs as shown by increased CFU during NSRCT (275.83) and restorative (249.83) procedures (Table 2). Our data demonstrate that adding 0.1% NaOCl and 0.2% CHX significantly reduced the total colony-forming unit as compared to that obtained from the baseline sample and positive control (Fig. 4). 0.1% NaOCl generated a mean CFU count of 92.17 thus performing better than 0.2% CHX that generated a mean CFU count of 146.83 during NSRCT (Table 2).

**Table 1:** Shows baseline CFU count in operatories 1,2, and 3 (negative control)

	Count (CFU)		
	Operatory 1	Operatory 2	Operatory 3
Base Sample 1.7 m – C1	136	121	129
Base Sample 1.7 m – C2	123	140	131
Average	129.50	130.50	130.00

**Table 2:** Shows the CFU count in operatory 3 when distilled water was used in DUWL

Sr. No		Endodontic Procedure	Restorative Procedure
1	Sample at 0.6m (A)	166	484
2	Sample at 2m (B)	227	184
3	Sample at 1.7m (C1)	206	204
4	Sample at 1.7m (C2)	446	236
5	Sample at 2m (D1)	281	190
6	Sample at 2m (D2)	227	201
Average		257.83	249.83

During class I cavity preparation, 0.1% NaOCl generated a mean CFU count of 85.83 as compared to 0.2% CHX which generated a mean CFU of 124.50 and the control group generated a mean CFU of 249.83 (Table 2). For the RP, a comparison of mean CFU scores in 3 different groups showed a statistically significant difference between the groups (f—8.574, *p* - .001) with the mean score of group 3 higher than group 2 followed by group 1 (Table 3). For the NSRCT, a comparison of mean CFU scores in 3 different groups showed a statistically significant difference between the groups (f—8.854, *p* - .003) with the mean score in group 1 being the lowest and group 3 being the highest (Table 3). The inter-group comparison of mean CFU scores in 3 different groups for restorative procedures shows a statistically significant difference between groups1and 3 (*p* - .003) as well as groups 2 and 3 (*p* - .023); but no significant difference between groups 1 and 2 (*p* - 1.00) (Table 4). The inter-group comparison of mean CFU scores in 3 different groups for NSRCT shows a statistically significant difference between groups1and 3 (*p* - .001) as well as groups 2 and 3 (*p* - .028); but no significant difference between groups 1 and 2 (*p* - 1.00) (Table 4).

**Table 3:** Shows the CFU count in operatory 1 when NaOCl was used in DUWL

Sr. No		Endodontic Procedure	Restorative Procedure
1	Sample at 0.6m (A)	68	128
2	Sample at 2m (B)	117	58
3	Sample at 1.7m (C1)	117	107
4	Sample at 1.7m (C2)	82	100
5	Sample at 2m (D1)	90	62
6	Sample at 2m (D2)	90	60
Average		92.17	85.83

**Table 4:** Shows the CFU count in operatory 2 when CHX was used in DUWL

Sr. No		Endodontic Procedure	Restorative Procedure
1	Sample at 0.6m (A)	126	156
2	Sample at 2m (B)	122	84
3	Sample at 1.7m (C1)	183	117
4	Sample at 1.7m (C2)	172	124
5	Sample at 2m (D1)	156	136
6	Sample at 2m (D2)	122	130
Average		146.83	124.50

### Distance of aerosol dissemination

DAs were found to disseminate as far as 3 m from the patient's head with the highest CFU count found at position A in all three groups and comparatively lesser counts at farthest distance D1 and D2 (Fig. 4).

### Statistical analysis

The data was entered and analysed using a statistical package for social sciences (SPSS) for Windows 26.0 (SPSS, Inc. Chicago, Illinois). The confidence intervals were set at 95% and a *p*-value < 0.05 was considered statistically significant. One-way ANOVA was performed and the Kruskal–Wallis test was used for intergroup comparison.

## Discussion

Since the Covid-19 pandemic, DA has gained significant attention [52]. Hence, several attempts are being focused to gain a better knowledge of the implications of DA. RP and NSRCT contribute to the majority of clinical work performed routinely in dental offices. Due to the close vicinity of dental treatment to the oropharyngeal region, the provision of dental care comes under scrunity [53–55]. There is a lack of specific clinical research information on restorative and NSRCT-generated DA and extenuation policies to lower microbial infection. To the best of the author's knowledge, this is the foremost attempt to compare the outcome of adding 0.1% NaOCl and 0.2% CHX in the DUWLs on the microbial count in DAs generated during RP and NSRCT. The findings of the present study showed that the addition of 0.1% NaOCl and 0.2% CHX in the DUWL significantly decreased the bacterial burden. Hence the second part of null hypothesis was rejected.

Although the NSRCT took a longer duration than the restorative procedure, there is no statistically significant difference in aerosolised bacterial generations in both procedures at all locations. We hypothesize the following:

- 1. Albeit the blood in the teeth treated with NSRCT acts as a strong carrier for microbial DAs, the use of intra-canal irrigants (NaOCl and CHX) may have contributed to bacterial inactivation in NSRCT versus the restorative procedure.
- 2. Here the “settle plate” method was used to evaluate the generation of DA over a period of 90 min. This unswervingly stops the survival of obligate anaerobes which might be found in inflamed pulp tissue [56].
- 3. Relatively lesser bacteria are present on the carious teeth compared to the inflamed pulp which needs pulpectomy.

The current study validates that RP as well as NSRCT generate aerosolized bacteria. Hence first part of the null hypothesis was rejected. The distance of DAs dissemination is a critical factor that contaminates the operatory. It was seen that a higher CFU count was obtained at the closest distance i.e. at 0.6 m compared to the rest of the positions. This was in accordance with results by Manarte-Monterio et al. who demonstrated that aerosol generation during restorative and endodontic procedures travelled a distance up to 10 feet or 3 m with statistically higher concentrations closest to the operator [57].

DUWLs are a source of water for patients' mouth rinsing, cooling high-speed rotatory devices and removing the debris formed during tooth preparation [28]. Numerous studies have shown that DUWLs tend to get severely contaminated [58, 59]. The majority of studies and research have reported the microbial contamination of DUWLs at levels of 1.5 × 10^2^ to 1 × 10^6^. Moreover, species like mesophilic bacteria, Legionella, and Pseudomonas aeruginosa were also reported in DUWLs [60, 61]. Therefore, contamination control is of vital significance in dental operatories. The studies have recommended flushing of DUWLs for 30–180 s in between patients during dental procedures [62]. However, conflicting results are seen regarding flushing of DUWLs indicating that flushing is a weaker mode of disinfection [31]. Yabune et al. found that microbes in the DUWLs were significantly reduced when an experimental tube was lined with Polyvinylidene fluoride [63]. But literature lacks any concrete information regarding the correlation between DUWLs lining and reduction in DAs.

In this study, pre-treatment and treatment protocols were standardized. Rubber dam isolation and use of high-vacuum suction were used to prevent the contaminated DAs from escaping the oral cavity [64]. Airflow circulation patterns in all three operatories were kept identical. A settle plate technique was used *i.e*. petri dish containing agar media was placed for a given period of time in order to allow the growth of aerobes and facultative anaerobes that are commonly observed in carious teeth [65]. This helps in quantifying the viable microbes that can settle, grow and propagate over the agar plate media. This sampling technique is commonly in dentistry [66].

In our study, the addition of 0.1% NaOCl in the DUWLs reduced the bacterial burden due to the strong antimicrobial activity of NaOCl. The effectiveness of NaOCl in disinfection depends upon the availability of free chlorine. When NaOCl is combined with water, it results in the formation of hypochlorous acid (HOCl) which is a feeble acid that dissociates into hypochlorite ion (-OCl) and proton (H +) that are effective against various pathogens [67]. These findings are similar to a previous study by Karpy et al*.* in which sustained NaOCl treatment was shown to improve the quality of water in DUWLs and decreased bacterial colonization [68]. However, there are a few potential concerns regarding the incorporation of 0.1% NaOCl in DUWLs. Its contact is not restricted to tooth alone even under rubber dam isolation. Nevertheless, American Dental Association has assigned 0.1% NaOCl as a safe antibacterial mouth rinse to be used directly on mucous membranes [69]. Moreover, when NaOCl reacts with microbial biofilm, then it leads to the formation of trihalomethanes as a by-product that has negative health effects such as cancer and adverse reproductive outcomes [70]. However, the levels of trihalomethanes formed are well below the Environment Protection Agency limits for portable water. The continuous and longer use of NaOCl has been demonstrated to corrode waterlines from electrochemical reactions [71–73]. But this can be eliminated by the use of lower NaOCl concentration with intermittent flushing of DUWLs with water free of chlorine [74–76].

Here, the use of CHX in DUWLs also showed a reduction in CFU count as compared to the control group. CHX acts on both gram-positive as well as gram-negative bacteria and it is bacteriostatic as well as bactericidal [77]. It binds to the negatively charged cell wall thereby altering the osmotic equilibrium. This was similar to the study conducted by Epstein et al.who showed a reduction in the bacterial count after treatment of DUWLs with CHX [78]. In dentistry, aerosols are generated in almost all the procedures. As stated above, these DAs are not only highly infectious, making dentistry a “very high-risk” profession but also spreads in all the directions to the greatest distance. Hence every attempt should be made to minimize the microbial content of these DAs. So, the current study is important that attempts the use of various chemical disinfectants to reduce the aerosolized bacteria.

## Conclusion

The addition of 0.1% sodium hypochlorite (NaOCl) or 0.2% Chlorhexidine(CHX) in DUWLs shows an effective reduction in aerosolized bacteria compared to distilled water. However, no significant difference between the antibacterial effect of NaOCl and CHX in DUWLs is seen. It is an efficacious and safe alternative to existing alleviation strategies for reduction in bacterial count by aerosol generation during endodontic and restorative procedures. Thus, it is a pragmatic clinical study that represents real-life scenarios and treatments with wider external applicability to the endodontic clinician community as well as general practitioners.

### Limitation

The study did not evaluate the spread of various aerosolized viral particles during endodontic and restorative procedures.

Future prospective randomized clinical trials are needed to study the effect of different variables on aerosol generation in each group.

## Data Availability

The data will be available on reasonable request from the corresponding author.
